# Health system determinants of tuberculosis mortality in South Africa: a causal loop model

**DOI:** 10.1186/s12913-021-06398-0

**Published:** 2021-04-26

**Authors:** Muhammad Osman, Aaron S Karat, Munira Khan, Sue-Ann Meehan, Arne von Delft, Zameer Brey, Salome Charalambous, Anneke C Hesseling, Pren Naidoo, Marian Loveday

**Affiliations:** 1 Desmond Tutu TB Centre, Department of Paediatrics and Child Health, Faculty of Medicine and Health Sciences, Stellenbosch University, Cape Town, South Africa; 2 TB Centre, London School of Hygiene & Tropical Medicine, London, UK; 3 The Institute for Global Health and Development, Queen Margaret University, Edinburgh, UK; 4 Tuberculosis and HIV Investigative Network (THINK), Durban, South Africa; 5 School of Public Health and Family Medicine, University of Cape Town, Cape Town, South Africa; 6 TB Proof, Cape Town, South Africa; 7 Bill and Melinda Gates Foundation, Johannesburg, South Africa; 8 The Aurum Institute, Parktown, South Africa; 9 School of Public Health, University of the Witwatersrand, Johannesburg, South Africa; 10 HIV Prevention Research Unit, South African Medical Research Council, KwaZulu-Natal, Pietermaritzburg, South Africa; 11 South African Medical Research Council-CAPRISA-HIV-TB Pathogenesis and Treatment Research Unit, Durban, South Africa

**Keywords:** Tuberculosis mortality, Health systems

## Abstract

**Background:**

Tuberculosis (TB) is a major public health concern in South Africa and TB-related mortality remains unacceptably high. Numerous clinical studies have examined the direct causes of TB-related mortality, but its wider, systemic drivers are less well understood. Applying systems thinking, we aimed to identify factors underlying TB mortality in South Africa and describe their relationships. At a meeting organised by the ‘Optimising TB Treatment Outcomes’ task team of the National TB Think Tank, we drew on the wide expertise of attendees to identify factors underlying TB mortality in South Africa. We generated a causal loop diagram to illustrate how these factors relate to each other.

**Results:**

Meeting attendees identified nine key variables: three ‘drivers’ (adequacy & availability of tools, implementation of guidelines, and the burden of bureaucracy); three ‘links’ (integration of health services, integration of data systems, and utilisation of prevention strategies); and three ‘outcomes’ (accessibility of services, patient empowerment, and socio-economic status). Through the development and refinement of the causal loop diagram, additional explanatory and linking variables were added and three important reinforcing loops identified. Loop 1, ‘Leadership and management for outcomes’ illustrated that poor leadership led to increased bureaucracy and reduced the accessibility of TB services, which increased TB-related mortality and reinforced poor leadership through patient empowerment. Loop 2, ‘Prevention and structural determinants’ describes the complex reinforcing loop between socio-economic status, patient empowerment, the poor uptake of TB and HIV prevention strategies and increasing TB mortality. Loop 3, ‘System capacity’ describes how fragmented leadership and limited resources compromise the workforce and the performance and accessibility of TB services, and how this negatively affects the demand for higher levels of stewardship.

**Conclusions:**

Strengthening leadership, reducing bureaucracy, improving integration across all levels of the system, increasing health care worker support, and using windows of opportunity to target points of leverage within the South African health system are needed to both strengthen the system and reduce TB mortality. Further refinement of this model may allow for the identification of additional areas of intervention.

## Introduction

In 2019, an estimated 360,000 people developed tuberculosis (TB) in South Africa; 58 % of these individuals were HIV positive, and 17 % died [1]. According to death certificates, TB remains South Africa’s leading natural cause of death [2], highest in HIV-positive people (particularly those admitted to hospital [3]), in whom it is often undiagnosed [4]. Morbidity and mortality after completion of antituberculosis treatment is also increasingly recognised as an important outcome [5] with individuals who ‘successfully’ complete TB treatment having a four-fold increase in mortality compared to the general population [6, 7].

Health systems are non-linear and are best thought of as complex adaptive systems, in which the many interacting parts make it near impossible to predict the behaviour of a system based only on the knowledge of its components [8]. The South African health system has undergone significant change over the past 26 years. Much has been written about the transition of health services after the end of apartheid and the importance of a freely elected government in driving health system reform [9]. From 1994, South Africa embarked on a series of strategies to reform primary health care (PHC) through orienting towards population health; focusing on health outcomes; developing integrated, efficient PHC teams guided by communities; establishing a district health system; and paying closer attention to the social determinants of health. [10] However, the effectiveness and quality of the health services remain sub-optimal, and health outcomes remain poor. Four colliding epidemics, including HIV and TB, and the corresponding disease burden in South Africa were well described in 2012 [11]. The public and private health sectors in South Africa have been described as ‘pro-rich’ although the poor bear the greater burden of ill-health (up to 13-fold increased incidence of TB) and have access to far fewer services compared to the rich [12]. A 2012 review identified differentials in social determinants of health, the need for comprehensive integration of all aspects of the South African health system, and improved surveillance and information systems as “core needs” [11] for the transformation of the South African health system.

Previous health system analyses of TB in South Africa have focussed on TB and HIV collaborative activities at facility level [13] as well as delays in diagnosis and treatment of TB patients [14]. Barriers to implementation of collaborative activities that have been identified included insufficient consultative leadership and political will; organisational culture; management, planning and power issues; unequal financing; and human resource capacity [13]. Factors associated with patient and health care system delays in diagnosis and treatment of TB included accessibility; staff skills and training; specimen and testing logistics; and the role of traditional healers [14]. Globally, weak health systems have been shown to impact on the performance of the TB care cascade[15] and calls have been made for monitoring beyond routine TB programme indicators to include the quality of TB care including clinical competence; timely, continuous, and integrated care; and respectful and patient-centered care [16]. However, while numerous studies have examined direct and clinical causes of TB-related mortality, health systems analyses have not been undertaken to explore the wider, systemic drivers of TB mortality. Using a series of systems tools, we aimed to identify factors underlying TB mortality in South Africa and describe how these factors relate to each other.

## Methods

### Context

South Africa is a high tuberculosis (TB) burden country [1] and has the largest HIV epidemic and antiretroviral therapy (ART) programme in the world [17]. The ‘front line’ of the South African health system is the almost 3,500 PHC clinics where a range of services are offered [18], including diagnosis and treatment for TB and HIV. TB diagnosis is currently largely reliant on passive case finding, where individuals presenting at PHC clinics are screened using the World Health Organization (WHO) four-symptom screen and sputum is evaluated using Xpert MTB/RIF (Xpert; Cepheid, Sunnyvale, CA). PHC facilities are grouped into a sub-district, and multiple sub-districts form a health district which is the level at which PHC services are managed. Across the 52 health districts of South Africa, the population density, demographics, and health outcomes vary [19]. Importantly the 2017 death rate during drug-susceptible TB treatment varied significantly across the districts from a low of 2.6 % to a high of 15.1 %; 9/52 districts had a death rate > 10 %; 8/52 had a death rate ≤ 5 % (including all 6 districts in the Western Cape Province); and 21/52 districts showed a decrease in death rate from 2016 to 2017 [19]. If an individual requires additional investigations and diagnosis, they will be referred from a PHC clinic to a district, secondary or tertiary level hospital. Hospitals may initiate TB and HIV treatment, but patients are then down-referred to PHC clinics for the continuation and completion of TB treatment. TB and HIV integration activities in South Africa have progressively been implemented and include testing all TB patients for HIV; screening all HIV-positive people for TB; and providing TB preventive therapy (TPT) to those who are eligible [20]. In South Africa, 93 % of TB testing in 2012 was conducted in the public sector at the National Health Laboratory Services [21] and, in the 2018 South African TB prevalence survey, 92 % of people with TB first accessed care in the public sector [22]. Almost all people diagnosed with TB are treated in the public sector with 3 % of TB medication in South Africa used in the private sector [23].

### South African TB think Tank

The South African National Department of Health established the TB Think Tank in 2014, drawing on national expertise, research capacity, and international networks, to support evidence-informed decision making, and was successful in supporting the first TB/HIV investment case for TB in South Africa [24]. The Think Tank has six task teams; in early 2020, the ‘Optimising TB Treatment Outcomes’ task team convened a meeting in Cape Town, South Africa, with 47 attendees included academics, clinicians, representatives of non-profit organisations, TB advocates, and TB programme officials (national, provincial, and district level). All members of the TB Think Tank were invited to submit abstracts on TB-mortality related studies which had recently been completed or were currently underway in South Africa at the time of the seminar. Abstracts were reviewed by three independent reviewers and scored according to whether: 1) the title reflected the study; 2) the study question was clear; 3) the methods were appropriate for answering the study question; 4) the results answered the study question; and 5) the discussion and conclusion were appropriate and suggested a way forward. In addition, to ensure diversity of perspectives, presentations from TB programme managers and implementing partners were included even if not scored as highly as those of academics.

### Systems Thinking

As described in more detail below, we used a systems thinking approach to identify factors underlying TB mortality in South Africa by: (i) understanding the context; (ii) using root cause analysis by individuals; (iii) group work to establish themes and consider relationships; and (iv) refinement of relationships and causal loop model development using a small core group.

Systems thinking provides the opportunity to understand, test, and revise our understanding of how the different components in a system work together and is increasingly used in public health [25]. TB mortality in South Africa was contextualised at this seminar through research-based oral and poster presentations. Attendees from the TB Think Tank included academics, TB programme managers, and non-governmental organisations, providing a diverse group at the workshop. Following the presentations, a facilitated discussion was conducted using four small groups. Workshop participants were allocated at random to groups and one workshop facilitator ensured diversity within each small group.

Systems thinking tools were described and facilitators for each group were available to assist with the implementation of activities. Each meeting attendee was asked to use the fish bone analysis and/or the “five whys” technique to identify the underlying health system reasons [26] why people in South Africa are dying of TB. Results were recorded on post-it notes and, after exhaustion of ideas, individuals worked in groups to produce *affinity diagrams* by organizing the post-it notes into logical groupings to identify strategic themes, [27] (referred to henceforth as *key variables*). Each group worked independently and identified 9–13 key variables which represented the underlying causes of TB mortality in South Africa. To challenge pre-existing mental models, groups then discussed relationships between each key variable and produced a visual representation of interactions between key variables (an *interrelationship diagraph*) using arrows to depict the ‘direction’ of each relationship [28].

Following the meeting, the authors reviewed the key variables in each group’s affinity diagram and interrelationship diagraph and undertook a series of meetings to aggregate the key-variables and document the relationships described. Based on the direction of the relationships in the interrelationship diagraphs, each key variable was assigned a score, which denoted the number of arrows moving towards and away from it. *Drivers* were those variables with the most arrows pointing away from them, *outcomes* had the most arrows pointing to them, and *links* had an equal number of arrows in and out [28]. A preliminary causal loop diagram (CLD) was developed to illustrate the causes of TB mortality in South Africa. By tracing the relationship between each variable in the CLD, the direction of change was described as the “same” or “opposite” and the effect of one part of the system on the remaining variables was then traced through each loop [28]. Loops can be classified as *reinforcing* or *balancing*. In a reinforcing loop, change in one variable results in the same change across the system and, eventually, has the same effect on the initial variable, perpetuating either an increase or decrease in the system overall [28]. In contrast, a balancing loop generates equilibrium: change in one variable results, eventually, in the opposite effect being exerted on the same variable following the effect across the system [28]. In a balancing loop an increase in one variable results in an eventual decrease in the same variable, producing a balanced state with no change. Through an iterative process the authors developed and refined the CLD using Vensim PLE for Windows Version 8.1 (https://vensim.com/).

### Ethics

This article reports the outcomes of a meeting of the National TB Think Tank mortality task team. As there were no human research participants, no formal interviews conducted, and data collected were not attributed to individuals, ethical approval was not sought. Opinions or viewpoints have not been attributed to specific individuals or groups of individuals and no identifiable details of meeting attendees are included.

## Results

**Fig. 1:**
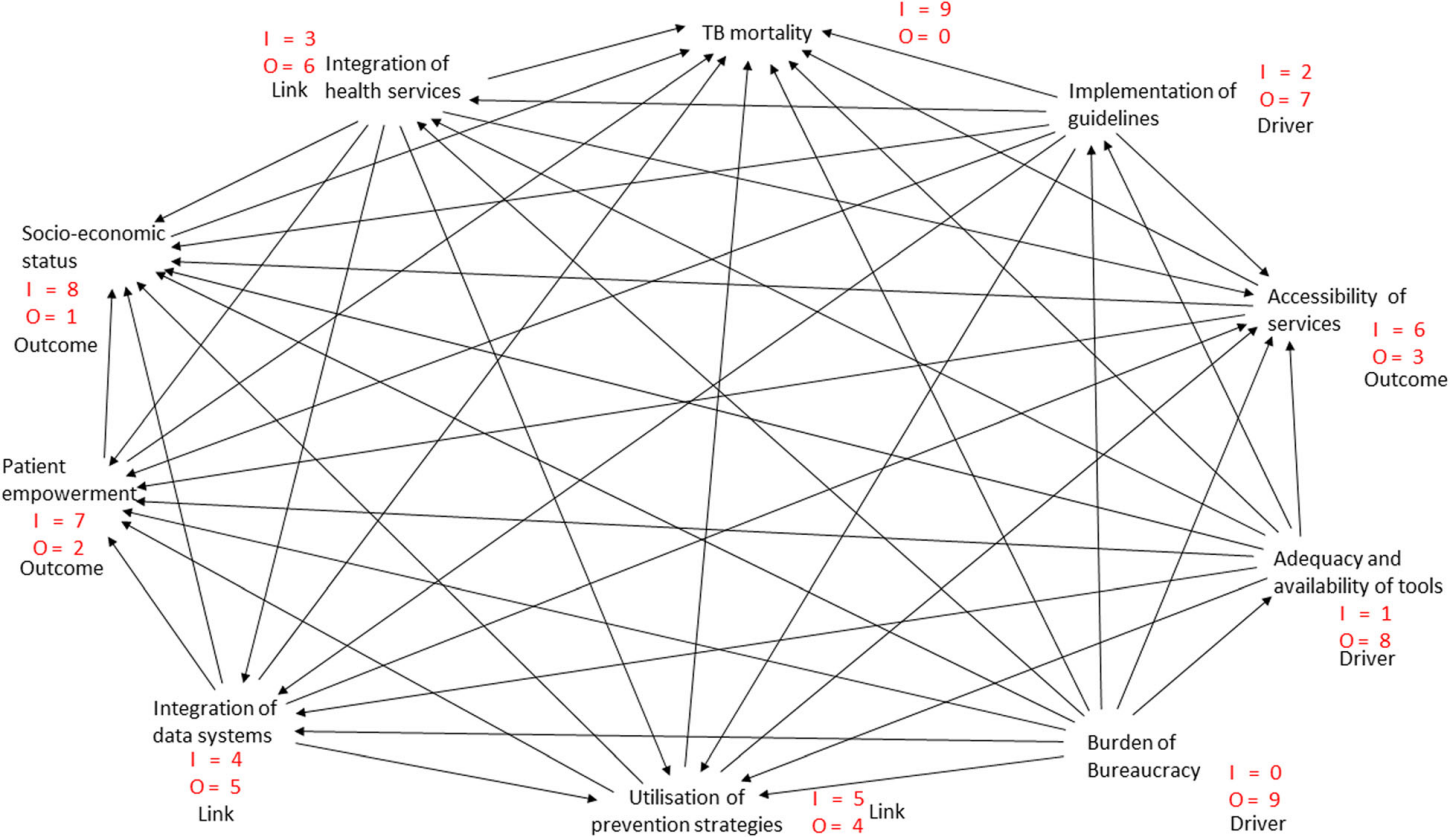
Interrelationship diagraph of key variables underlying TB mortality in South Africa, depicting drivers, links and outcomes. I: in; O: out; TB: tuberculosis

**Fig. 2:**
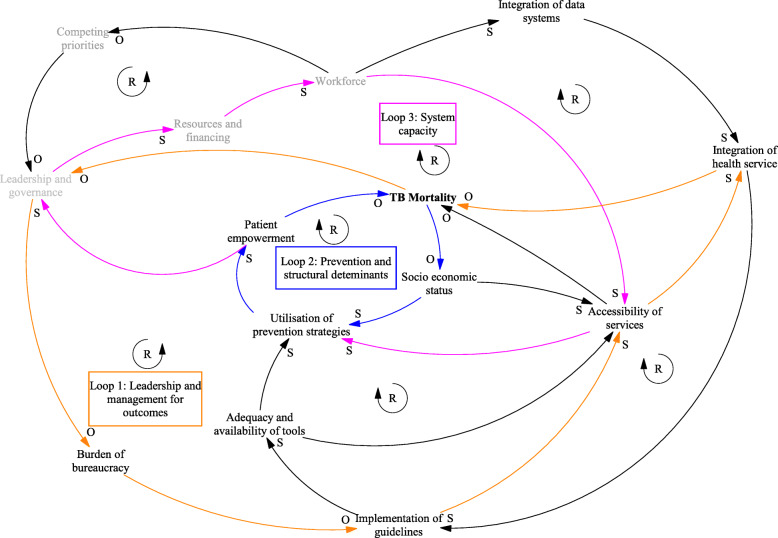
Causal loop diagram describing factors affecting TB mortality in South Africa, developed through group process. O: opposite; R: reinforcing; S: same; TB: tuberculosis. Variables in black reflect the key variables identified and variables in grey reflect additional explanatory variables that were added. Three colours were used to differentiate the reinforcing loops discussed in this paper, orange: Loop1; blue: Loop 2; purple: Loop 3

Nine key variables relating to TB-related mortality in South Africa were identified; three key drivers: *adequacy and availability of tools, implementation of guidelines*, and *burden of bureaucracy*; three links: *integration of health services, integration of data systems*, and *utilisation of prevention strategies;* and three outcomes: *accessibility of services, patient empowerment*, and *socio-economic status* (Fig. 1). Through the iterative process of developing the CLD, four further explanatory variables were added: *workforce*; *resources and financing*; *leadership and governance*; and *competing priorities*. These were included in the CLD and provided clarity on the mechanisms through which the key variables interacted (Fig. 2).

### Drivers

The *adequacy and availability of the latest tools* for TB screening, investigation, prevention, diagnosis, treatment, and adherence were discussed. Appropriate guidelines and algorithms for the comprehensive management of TB were widely recognised as important to reduce mortality, but gaps in implementation, quality of services, and evaluation of policies were identified as contributing to mortality. In addition, limited support for the *implementation of guidelines* for complex disease, comorbidities and drug resistant TB were also highlighted. The significant number of administrative procedures associated with TB management, which, ironically, compromises the quality of care it seeks to safeguard, was labelled as the *burden of bureaucracy*.

### Links

Multiple *components of service integration* were described: the lack of public and private health service integration for TB testing and treatment; poor referral pathways between different levels of care, facilities, and districts; poor integration of TB into different programmes such as HIV, reproductive health, and non-communicable diseases; and the inability of the system to provide a TB patient access to all these services during a single visit. The failures of TB data systems to communicate, exchange data, and use health and related data in a timely manner were identified as contributing to TB related mortality. The inability to ascertain results, treatment history, and outcomes of patients regardless of place or time of management was highlighted, with the assertion that poor intersectoral integration and lack of data sharing between health, social and correctional services further undermines efforts to comprehensively manage TB patients. The failures of the optimal use of proven *TB prevention strategies* also contributed to TB related mortality including the coverage of *bacille Calmette-Guérin* (BCG) immunisation at birth (and the lack of effective alternatives more than a century after its development); poor tracing, screening, and follow-up of contacts; neglect of infection prevention and control (IPC) measures in public and healthcare settings; and failures in the provision and uptake of TPT. Given the important role of ART in reducing TB-associated morbidity and mortality in people with HIV, strategies to prevent and treat HIV were also considered TB prevention strategies.

### Outcomes

Limitations to *accessibility of services* included availability, affordability, and acceptability of TB services [29, 30]. This definition of access has been presented previously and allows consideration of true access rather than coverage of health services as a proxy for access [30]. Availability (physical access) referred to services being available to meet the needs of the population and included geographical location, operating hours, and actual services supplied. Affordability (financial access) referred to the full costs to the health service user, including transport costs and loss of income whilst travelling to and utilizing the health service. Acceptability referred to expectations of the health system user, health care worker (HCW) attitudes, waiting times, and the way the service is organized (as experienced by the patient). Earlier work reported that the greatest deficiency in access to health services in South Africa was availability: constraints of affordability related mostly to travel costs, while acceptability levels were high, at 90 % [31]. Importantly, rural households and vulnerable populations, including those who were less educated, poor, and unemployed tended to be less likely to have adequate access [31]. While health service acceptability has generally been high in South Africa [31], at our workshop, attendees described TB preventive therapy as a strategy that was perceived as unacceptable in many communities. Specific strategies, including TB prevention, need to be evaluated for acceptance across the diverse cultures in South Africa. The lack of *patient empowerment* included the way patients understood their role and the limited knowledge and skills patients were given by their health care providers. This disempowerment is further entrenched by a health system which fails to recognize community and cultural differences or encourage patient participation. We also considered patient empowerment to include TB-related stigma and the ability of individuals to manage the additional burden that stigma added to their lives. The overall *socio-economic status* of individuals with TB was identified as an underlying cause of death among people with TB in South Africa. This determines patients’ access to resources (primarily financial resources, but also educational, as well as well-structured and ventilated housing, opportunities for employment, transport, and security); vulnerability and exposure to substance abuse; and appropriate additional support (nutritional, mental health services, or social care).

*Workforce; resources and financing; leadership and governance; and competing priorities* were additional explanatory variables thought to have an important impact on mortality through interactions with other elements. *Workforce* included staff capacity, attitude, and burnout. *Resources and financing* referred to how funds are allocated, managed, and spent at national, provincial, district, sub-district, and facility levels; the processes for motivating for additional funds; and how funding was re-allocated. Effective *leadership and governance* across every level is required and we considered the importance of different leadership styles. Transactional leadership has been considered to be the most prevalent model in existing health systems [32, 33] and requires those in leadership positions to effectively plan, implement, monitor activities and reward employees [32, 33]. Adaptive leadership is used to enable groups of people to overcome change, and the adaptive leader typically does not focus on the technical solution but rather the process of collaboration to overcome old values and beliefs empowering teams to develop their own solutions [32, 33]. Transformational leaders inspire teams to look beyond individual motivations and work towards the organisational mission but are dependent on the vision and values of the leader [33]. Earlier work has argued that servant leadership is the ideal within health systems as the leader remains focused on the needs of others, both healthcare workers and patients [33]. The servant leader uses the strength of the team to develop trust and core moral and ethical beliefs to effect changes and improve the value of care that is delivered by the health system [33]. The greatest limitation with all these models of leadership is the reliance on an individual as a leader. Participatory leadership built on the value of multiple perspectives and diverse strength embodies collective decision making to effect change across the health system and includes ‘interactive’, ‘collective’, ‘consultative’, ‘distributed’ and ‘horizontal’ leadership [34]. A recent series on health leadership in Africa showed the negative impact of existing leadership styles and the potential for change using new forms of participatory leadership which values the role of health system actors and enables greater participation in leadership [35]. *Competing priorities* included urgent or emergent conditions that have undermined TB service delivery across all levels of the health system, with multiple programmes competing for limited resources.

### Causal loop diagram

A causal loop diagram was constructed to illustrate the relationships between the key variables and TB mortality; three distinct reinforcing loops were described (Fig. 2). *Loop 1*, ‘*Leadership and management for outcomes’*, is a reinforcing loop from leadership through to TB services and mortality. *Loop 2, ‘Prevention and structural determinants’* is a reinforcing loop affecting TB mortality, including the utilisation of TB prevention strategies, patient empowerment, and socio-economic status. *Loop 3, ‘System capacity’* includes the role of leadership and the workforce in improving the accessibility of services and the implementation of TB prevention strategies. In loop 3, the effect of the use of prevention strategies on patient empowerment and leadership completes this reinforcing loop, where we expect empowered patients will contribute to improved leadership through elements of participatory leadership.

## Discussion

Using an iterative process, a series of systems thinking tools, and drawing on a wide range of experience, we have presented a conceptual model of TB mortality within the complex South African health system. This provides an opportunity to consider the key variables, their interactions, and the points of leverage for intervention to reduce TB mortality.

### Loop 1: Leadership and management for outcomes

This vicious cycle runs from poor leadership, through the burden of bureaucracy, to reduced accessibility of TB services, which worsens outcomes (TB-related mortality) and therefore worsens leadership through negative reinforcement. Several studies have documented the impact of health systems performance on treatment outcomes with an increased likelihood of unsuccessful health outcomes when health systems are dysfunctional [36, 37]. A 2012 review recognised the new political leadership in South Africa since 1994 and the proactive policy changes, but these were undermined by managerial bureaucracy [11]. The National TB treatment guidelines call for a sensitive and supportive approach to patients’ needs, but still specifically include directly observed treatment, which requires a treatment supporter to watch the patient swallowing tablets [38]. The legacy of the TB programme and bureaucratic delays and resistance to change have meant that the decisions made at various levels of the health system lead to failures in implementation of appropriate and available TB management guidelines, poor use of available tools, and limited access to TB services, which contribute to delayed diagnosis, sub-optimal treatment, poor TB treatment outcomes, and increases in TB-related mortality [39, 40]. Barriers to access also undermine efficacious treatment and failing to complete treatment can lead to drug-resistant disease [41–43], which has worse outcomes [44] and high mortality rates [45]. Supportive supervision and accountability [37], together with the availability and implementation of guidelines and protocols [46, 47] improve quality of care and health system performance, thereby reducing TB mortality and disease burden [37, 46, 47]. An opportunity exists for improving leadership in South Africa. Participatory leadership can be used to facilitate collaborative work to empower teams to develop efforts to reduce the burden of bureaucracy and to take actions to convert loop 1 to a *virtuous cycle*, where positive change will perpetuate further positive change. When considering the historical emphasis on transactional leadership, participatory leadership can be used to engage academics, clinical staff and health managers to jointly set targets for the implementation of changes [33] within the TB programme. A strength of servant leadership is its alignment with the values of healthcare workers and the delivery of patient-centred care [33] and these principles should be part of participatory leadership.

In the South African context, where almost 60% of people with TB are HIV positive [48], integration of TB and HIV services is important to improve accessibility and reduce barriers to care. [49]. Evidence supports the feasibility, safety, and effectiveness of integration of ART and TB services, [50], while failing to integrate TB and HIV services has been shown to increase mortality and poor treatment outcomes [51, 52]. Greater overall integration is important for reducing TB mortality, be that integration between HIV, TB, and noncommunicable disease programmes [53] or integration across levels of service. Recent cohort studies have highlighted the relationship between poor follow-up and hospital-readmission and mortality [54, 55]. Improvements in patient pathways between PHC facilities and hospital are essential, particularly for people living with HIV. Public-private collaboration has been identified as a promising strategy for TB control globally, but limited projects have been implemented in South Africa [56] and may reflect a lower priority as the vast majority of TB is diagnosed and managed in the public sector [21–23]. Comprehensively addressing integration of services has the potential to provide TB patients with a continuous service at PHC following the in-hospital diagnosis of TB; a reduced time between diagnosis and treatment support in a PHC setting; and a better link of patients to community-based services following a diagnosis of TB.

### Loop 2: Prevention and structural determinants

The relationship between TB mortality, socioeconomic factors, access, and treatment practices has been well established [57] and in this loop we illustrate the complex ways socio-economic status relates to TB mortality through the uptake of routine prevention strategies and patient empowerment, and how increasing TB mortality further perpetuates inequalities in socio-economic status. Interventions to address social and economic factors of individuals have included small incentives or food support but have not addressed the underlying health inequalities which drive the ability to access and sustain successful TB treatment [57]. Interventions to address socioeconomic inequities within this loop require intersectoral collaboration; addressing the social determinants of health is critical if we are to reduce TB mortality in South Africa [58]. Prevention strategies, defined as the range of measures intended to interrupt transmission and reduce incidence of TB and HIV, have considerable influence on TB mortality by their effect on the burden of disease in the population, [59] but socio-economic deprivation affects an individual’s ability to make use of these prevention strategies. Addressing the sub-optimal implementation of prevention strategies [60–63] requires a health system-level approach to improve the uptake of TB prevention [64] and provide a bridge between guidelines and utilisation in practice [62, 65]. A window of opportunity exists as South Africa plans for the roll-out of revised TB prevention guidelines in early 2021. These guidelines aim to expand the eligibility criteria for TB prevention to reach everyone with significant exposure to TB and offer multiple options for the duration of preventive therapy from 3 to 12 months. Creating the appropriate demand and increasing the utilisation of TB prevention has the potential to improve patient empowerment as communities are given the opportunity to engage with TB prevention beyond the narrow focus of previously defined at-risk populations (children under 5 years of age and people living with HIV). Patient empowerment is a fundamental component of participatory leadership [34]. Global and local responses to HIV including the Treatment Action Campaign and Section 27 have demonstrated how patient empowerment and the development of advocacy groups were used to guide health leaders on treatment policy and the allocation of financial resources [34]. The opportunity for leveraging the benefits of participatory leadership in the South African health system can be seen in Loop 2 where an increase in empowered patients could contribute to improved leadership and governance and generate a virtuous cycle.

### Loop 3: System capacity

*‘System capacity’*, here, is not restricted only to the ability of HCWs to provide the best care but rather reflects the overall health system’s capacity to create and foster the right conditions for optimal care. We described how existing poor leadership and limited resources have compromised both the workforce and the performance and accessibility of TB services. This negative effect further disempowers patients. In South Africa, we have seen how empowered patients have demanded more from health leaders. The Treatment Action Campaign launched in 1998 to campaign for access to HIV treatment achieved a South African Constitutional Court ruling ordering the provision of antiretroviral therapy to mothers for the prevention of mother to child transmission of HIV in 2002 [66] and was integral to the expansion of the ART program. Loop 3 suggests through the strategy of TB prevention there is an opportunity to develop leaders who are accountable and responsive to the needs of the people. Earlier work has shown how newly introduced, additional services are likely to be the first to be compromised when resources are constrained and the burden on the workforce is increased [67]. Consideration of HCW capacity provides an opportunity to increase the numbers of well trained (knowledge and skills) and motivated staff. HCW capacity-building needs to move beyond clinical education to include skills for broader population health management, patient-centred care, patient counselling, cultural sensitivity, and understanding and managing bias and stigma [16]. It is critical that this takes place across the wider health system, as people with TB make contact with a range of staff, not just specialised TB staff in TB services. When combined with good supervision and support, trained and supported staff have the distinct advantage of improving health outcomes for patients [68]. Lessons from Lesotho have shown how capacity building with training and support can be used to strengthen the implementation of a complex health intervention like the management of multidrug-resistant TB [69]. This contrasts with early experiences from South Africa, where HCWs experienced the drug-resistant TB programme with fear and anxiety, having concerns around being unsupported and the quality of care they could offer [70]. The National Department of Health should prioritise the ongoing support and development of HCWs, recognising the critical place of capacity development of all staff and its potential to affect TB mortality.

Leadership, resources, the attention of the workforce, and accessibility of facilities have been compromised in 2020 due to the coronavirus disease (COVID-19) pandemic and this has resulted in unprecedented drops in screening and testing of people with possible TB [71]. Despite this, there are opportunities afforded by the response to the pandemic, including respiratory protection and IPC, that can be positively leveraged for TB. Public-facing TB dashboards (highlighting contagion and loss of life in specific geographic areas); communication campaigns; and real-time accountable performance monitoring of TB services, as implemented for COVID-19, may improve patient empowerment and integration of services [72].

### Strengths and limitations

The underlying factors identified represent a wide array of experience in the field of TB in South Africa. The use of individual brainstorming for generating ideas and group work for organising the potential key variables created the opportunity for all attendees to contribute, drawing on diverse and experienced perspectives. We have produced a plausible model and provided evidence for these relations from the published literature. We were unable to validate our model but propose that further health system research could identify opportunities for intervention and support the development of local action plans to be implemented and observe the changes in our model. Previous work using group model building and a CLD allowed the development of local action plans by key stakeholders to improve the poor quality of maternal health services in the Eastern Cape [73]. In Rwanda, following the national scale-up of integrated community case management of malaria, pneumonia, and diarrhoea a health systems study and CLD was used to identify determinants of sustainability of the intervention and potential risks [74]. This presents the opportunity for future work using our CLD to develop local action plans for implementation and consider strategies for mitigating possible future risks as in the preceding studies described.

## Conclusions

We describe underlying health system factors contributing to TB mortality in South Africa. We have presented a CLD to illustrate their interactions and describe three important reinforcing loops, ‘Leadership and management for outcomes’, ‘Prevention and structural determinants’, and ‘System capacity’. Our analysis highlights the complex drivers and multiple interactions within both the national TB programme and the wider health system that contribute to the continued high levels of TB-related mortality in South Africa. Interventions to address mortality will be of limited effect unless they are designed and enacted with consideration of the constraints and dynamics of the system. Cross-cutting measures that look to strengthen the overall system include the call for improvements in leadership with a reduction in bureaucracy in the health system. The aspirational goal of participatory leadership has been highlighted as have been the opportunities to uses some of the strengths of other leadership styles. Additionally, improved integration across all levels; and a focus on increasing HCW capacity and support were highlighted. Using windows of opportunity, such as those presented by COVID-19 or the release of new guidelines, open up the possibility of leveraging small efforts to effect significant change in the health system. Further refinement of this model may provide additional opportunities to identify points of leverage to reduce TB mortality and should be evaluated.

## Data Availability

Data sharing is not applicable to this article as no datasets were generated or analysed during the preparation of this manuscript.
